# Development of advanced progress recognition algorithms for construction monitoring

**DOI:** 10.1371/journal.pone.0333262

**Published:** 2025-10-09

**Authors:** Lai Yingdong, Lin Zhijun, Ye Zhijie, Zhang Jun

**Affiliations:** 1 Jiangmen Power Supply Bureau of Guangdong Power Grid Co., Ltd., Jiangmen, Guangdong, China; 2 Infrastructure Department of Jiangmen Power Supply Bureau, Guangdong Power Grid Co., Jiangmen, Guangdong, China; 3 Taishan Power Supply Bureau of Jiangmen, Guangdong Power Grid Co., Ltd., Jiangmen, Guangdong, China; Islamic Azad University Mashhad Branch, IRAN, ISLAMIC REPUBLIC OF

## Abstract

**Introduction:**

The traditional methods of Construction Progress Monitoring (CPM) involve manual inspection and reporting, which are slow, error-prone, and labor-intensive.

**Purpose:**

This study aims to introduce a novel, automated approach for CPM using YOLOv8, a state-of-the-art object detection algorithm, to enhance efficiency and accuracy in monitoring construction projects.

**Methodology:**

YOLOv8 is employed for its real-time processing capabilities and high precision, making it suitable for identifying and tracking construction elements in images and videos captured on-site. This study creates a comprehensive dataset of construction images and videos to assess and validate the proposed method with meticulous labeling of relevant objects.

**Results:**

A custom-labeled dataset of 768 images of window installation stages was developed and used to train the model. The proposed YOLOv8 model achieved a mean Average Precision (mAP@50) of 0.953, mAP@50–95 of 0.678, precision of 0.91, and recall of 0.88. This integration of computer vision into CPM offers substantial benefits, including reliable, efficient, and cost-effective progress monitoring.

**Innovation:**

This approach presents an innovative computer vision application in construction progress monitoring. It facilitates timely decision-making throughout the project lifecycle and offers a practical alternative to manual CPM methods. Using YOLOv8 for automated CPM is a novel contribution to construction project management, potentially impacting the successful completion.

## 1. Introduction

The timely completion and cost control of the power line construction projects depend on effective progress monitoring. As the complexity of these projects increases, accurate and efficient tracking becomes essential. The commonly used approaches for performance evaluation of this type of project mainly depend on checklists, inspections, and reviews that require on-site construction observation and the status and progression at specific times. These commonly used approaches are time-consuming, require a large workforce, and are prone to errors that cannot be avoided. This causes unplanned delays in the project or may cause a general downturn in the performance level. Furthermore, the manual methods are subject to human factors, which may cause biases in the collected information.

Effective CPM is widely recognized for enhancing the quality of physical assets in the Architecture, Engineering, Construction, and Operations (AECO) industry through effective and systematic quality management. The quality of the work being put into the buildings plays a critical role in the buildings’ performance, which is necessary for delivering advanced organizational solutions like energy service contracts [1], predictive building automation and control [2], and performance-based maintenance [3]. The primary investigations in digital CPM focus on utilizing mobile applications installed on mobile and wearable devices at construction sites, as well as creating construction business process models [1,4]. However, a recent trend exists to use technological advancements for automated CPM [5].

Previous methods, such as image processing, laser scanning, and Building Information Modeling (BIM) [6], and semantic web technologies [7,8], all have limitations. The image-based procedure generally requires laborious off-site digitizing, as well as laser scanning and BIM, which are costly and time-consuming. Furthermore, semantic web technologies and the existing commercial solutions are complex. These limitations highlight the need for efficient, more accurate, and low-cost CPM solutions. This is essential for the use of deep learning [9] and Computer Vision [10], which is relevant to the automated CPM with object detection mechanisms. These algorithms can detect and follow objects from images and video footage that may be taken using airborne devices such as drones. These are relevant when determining the construction progress evaluation of power lines. Data on the coordinates of objects’ location, size, and orientation can be used to prepare progress reports in real-time, informing the project managers immediately and highlighting changes to the project’s progress.

This study focuses on monitoring the Windows installation process as the primary case study for tracking construction progress and analyzing results quickly, enabling appointed Quality Managers to notify the property owner and contact the main contractor to rectify unacceptable installations. This study proposes a novel automated CPM technique using the YOLOv8 object detection technique. The proposed approach can successfully detect construction items from site photographs using YOLOv8’s real-time recognition ability with high accuracy. As a one-stage object detection algorithm, YOLOv8 achieves better accuracy and performance compared to other object detection models. Its structure enables the detection of multiple objects simultaneously and is highly effective in addressing the real conditions encountered on construction sites.

This study will investigate the feasibility of using YOLOv8 as an automation tool for construction progress monitoring and provide a method to facilitate more efficient and accurate CPM by focusing on detecting and tracking key construction elements, including materials and equipment. The custom dataset compiled from actual construction projects allows for a realistic assessment of the model’s capabilities in a real-world setting. Unlike previous studies that primarily focused on accuracy or F-measure, this study introduces and evaluates alternative performance metrics that highlight the efficiency of YOLOv8 in construction site environments, which could reduce the need for manual labor, minimize delays, and improve project management efficiency.

## 2. Literature review

Over the years, there has been increased concern about applying computer vision (CV) for construction site surveillance. The early work focused on using CVs in the Architecture, Engineering, and Construction (AEC) sector (20). A plug-and-play implementation of object detection, tracking, and activity recognition in real-time site monitoring has further boosted the usage of CV techniques [11]. The role of a CV extends beyond progress monitoring in construction work; studies have been conducted to analyze its use in identifying safety risks in construction sites, highlighting its importance in promoting safety and efficiency simultaneously [12]. Furthermore, a study has been conducted on applying CV methods in creating 3D models of construction sites using Structure from Motion (SFM) and photogrammetry approaches concerning on-site image and video data [13]. Although these applications differ from our focus on real-time monitoring progress in power line construction, CV’s adaptability and potential to revolutionize the construction industry are highlighted in the technology.

The study presented the recent CV innovations for construction site surveillance [14] and methodologically reviewed the automatic object recognition methods, creating a remarkable background for enhanced progress check technology. Similarly, the authors [15] have presented Intelligent monitoring systems involving AI, with the integration of CV algorithms reported, focusing on progress tracking, safety assessment, and resource management in construction projects. This aligns with our objectives of real-time object detection for monitoring the progress of the power line using aerial data. In addition, at the time of the most recent synthesis of object detection/ tracking in construction, the work in [16] has listed several critical issues that should be focused on to enhance monitoring efficiency.

Thus, CV-based approaches can be improved for the progress monitoring of power line construction by integrating computer vision and BIM, which contributes to the digital transformation of construction site management. While earlier CPM approaches have employed computer vision tools such as Mask R-CNN or object tracking for element detection [5,13], these methods often lacked real-time processing capability or seamless integration with BIM environments. In contrast, the YOLOv8-based system enables real-time detection of windows across installation stages, enhanced with QR code recognition for precise localization and synchronization with BIM models [17,18].

With the modern-day advanced tracking of construction progress, new advancements have embraced the use of YOLOv8 and kindred deep learning instrumentation to enhance the level of accuracy and speed at which things can be identified and utilized in real-time applications. The authors propose a real-time CPM system utilizing YOLOv8 to monitor construction objects, asserting that the method’s effectiveness, as measured by precision, recall, and F1-score, is enhanced by significant improvements over manual surveys [19]. In complex site experimental conditions, achieving ~94−92% accuracy, Wu et al. present the strategy to add CBAM, GAM, and SIoU loss as enhancements to the YOLOv8-CGS to detect helmets effectively [20]. A multi-task framework was introduced to extend the YOLOv8 for detection, segmentation, pose estimation, and tracking, enabling comprehensive surveillance of safety on construction sites [21]. The authors of this article developed the CIB-SE-YOLOv8 model, where SE attention and C2f blocks are used to improve the accuracy and efficiency of real-time detection of PPE [22]. A YOLOv8-based system that tracks the bricklaying process and incorporates detections into a 3D BIM digital twin can serve as an example of your progress tracking system [23]. Exploring the problem of small objects detection, the SO-YOLOv8 attempts to enhance the detection of small-scale components on the sites, which is a serious problem in construction settings [24]. Park et al. demonstrated both the low cost and good performance of an edge-device risk detection system based on YOLOv8, which yields a real-time result with ~80 mAP and is technically realizable at remote locations [25]. Moreover, Zhang et al. proposed a safety algorithm based on YOLOv8, aiming to detect PPE and hazardous zones, which shares a similar methodology with transfer learning and custom training sets [26]. In a more general construction setting, Qi et al. looked at the integration of knowledge graphs and computer vision to provide a better way of tracking the progress of concrete bucket pouring [27]. Finally, the Safe-Construct framework is the first to explore 3D multi-view violation recognition on synthetic data, advancing the state of the art in spatial situation understanding in safety surveillance [28].

In computer vision, object detection stands as a cornerstone technology. This task involves pinpointing and classifying specific objects within images or videos [29]. Its applications span numerous industries, including autonomous vehicles [30] and robotics [31], and are increasingly relevant to our study, i.e., construction progress monitoring. The advent of Convolutional Neural Networks (CNNs) has significantly enhanced the domain of object detection due to their feature extraction capabilities. It excels at automatically learning intricate features directly from raw pixel data, leading to enhanced accuracy and improved generalizability in object detection tasks [32,33]. This paves the way for highly effective real-time object detection in power line construction progress monitoring using CV, as explored in this study.

Real-time processing is paramount for effective construction progress monitoring. Fortunately, the latest versions of YOLO object detection algorithms can make faster computations, achieve competitive accuracy, and incorporate advancements that refine both aspects [24]. These architectural improvements and optimization techniques empower YOLO to deliver superior performance, making it particularly suitable for our study on real-time object detection in power line construction projects.

## 3. Methodology

### 3.1. Research flowchart

**Fig 1:**
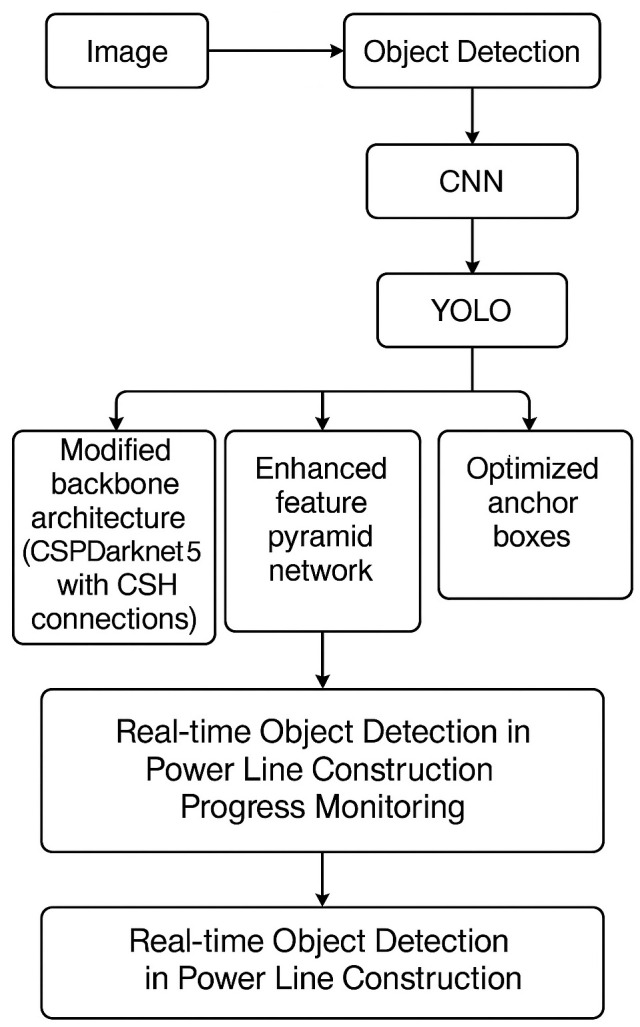
Research Flowchart.

Fig 1 *is presented the research flowchart*.

### 3.2. Object detection algorithm

The use of YOLO in this study is based on the following aspects:

- A modified backbone architecture based on CSPDarknet53 with Cross Stage Hierarchical (CSH) connections,
- An enhanced feature pyramid network for improved handling of objects with varying sizes and aspect ratios,
- Optimized anchor boxes tailored to the specific object classes and aspect ratios present in the training dataset and
- The Mosaic data augmentation technique exposes the model to diverse object scales, orientations, and lighting conditions. Additionally, it employs an improved loss function for more accurate and stable training and efficient implementation using CUDA and CUDNN libraries.

### 3.3. Object-identification for window installation

In the context of CPM and quality management, the documentation of window installations is vital for enabling stakeholders to assess ongoing work visually [34]. Timely and accurate CPM for Windows is essential to ensure that construction activities progress according to schedule and quality requirements, including the prevention of ’hidden’ errors and omissions [35].

Enhanced accuracy in object detection facilitates advanced capabilities such as identifying missing parts, determining the correct window type, and verifying assembly tolerances. Implementing object detection algorithms can improve safety by automating visual documentation through integrated devices or UAVs, replacing manual monitoring processes. This reduces the risk of accidents and injuries associated with quality management tasks in complex construction sites. Outdoor object detection for windows employs a cautious approach due to the challenges posed by polished or glass surfaces, which can confound detection algorithms. Window frames often exhibit limited distinguishing features for detection and classification. Moreover, variations in visual attributes, such as the color of sealing tapes during installation, can introduce errors in fully automated detection processes. A unique QR-code sticker is affixed to each window to overcome these issues. This activity is because QR codes can be robustly detected and located, providing sufficient image quality. An active learning approach is envisaged for the outdoor detection of installed windows. The details of the settings are provided in Table 1.

**Table 1:** An active learning approach for the outdoor detection of installed windows.

Feature	Indoor	Outdoor
	**General Features**	
Parties involved	4	6
Access to Quality Mgr.	yes **Object-Identification Feature**	scaffold required
Parts for identification	handles, hinges, actuators, etc.	nearly none
Obstructions	Limited	scaffold, power lines
Objects in the background	depends on the site	multiple
Mirroring effect	Limited	high, due to reflective surfaces

### 3.4. Algorithm benefits

**Fig 2:**
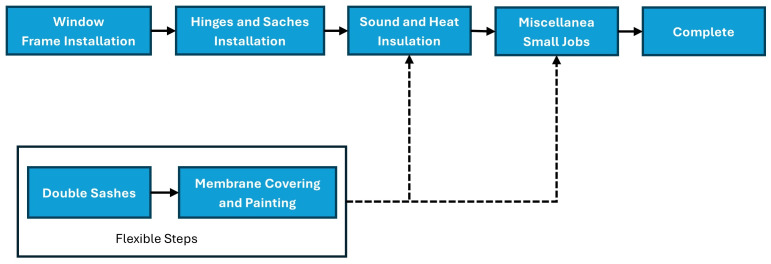
Installation Process of a Window.

- Automatic detection and location of windows using real-time processing, eliminating the need
- For manual checks.
- Ability to obtain current information on the progress of all window installation steps (potentially referencing a figure, Fig 2, not shown here).
- Facilitation of proactive decision-making by identifying any deviations from the expected
- Installation process.
- Rapid personnel identification requires corrective actions for any issues.
- Providing real-time information to relevant personnel through electronic means.

### 3.5. Dataset creation and annotation

The success of this study hinges on a strong foundation of data. Data collection is the essential first step, providing raw material for training the object detection model and making accurate predictions. A comprehensive data set comprising high-quality images is crucial to achieving reliable results. In essence, the quality and quantity of data directly influence the accuracy and trustworthiness of the final object detection model.

#### 3.5.1. Indoor object identification scenario.

To ensure the quality and relevance of our dataset, we collected 347 high-resolution JPG images (3060 × 4080 pixels) from the Beyer-Bau construction site in January 2023, from 8:00 a.m. to 12:00 p.m. The dataset is divided into three installation stages: pre-installation (105 images), partial installation (138 images), and complete installation (104 images). The environmental conditions varied across images, including overcast lighting (182), sunny conditions with glare (91), and indoor lighting (74). Window types include both single and double-sash frames. The dataset captures a variety of construction scenarios, with a focus on window construction, to support future monitoring efforts. To enhance the model’s generalizability, images were taken under varying conditions, including window types, camera angles, lighting, and diverse foreground and background elements. This comprehensive data collection is fundamental to the effectiveness of the proposed approach.

**Fig 3:**
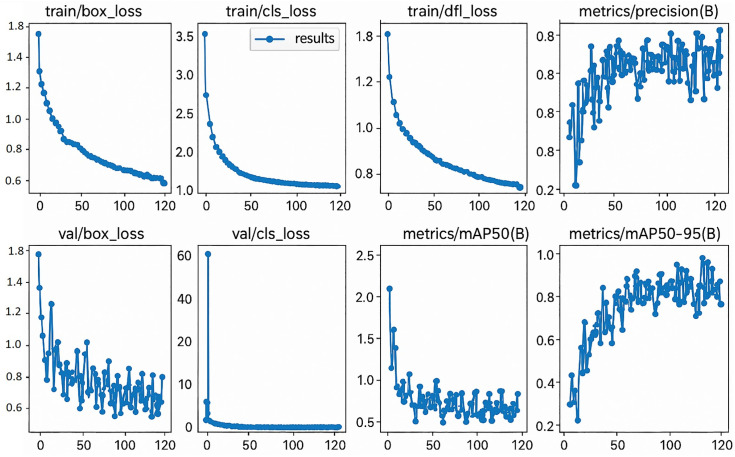
Various metrics of the training model.

By analyzing the project requirements, it was observed that there is a dire need to track both the completion percentage and the order of operations for window installation. Fig 3 details the project process from the beginning to the end, dividing it into six stages with corresponding completion percentages. However, the process requires additional flexibility to handle situations where the order of steps can vary. This is particularly relevant for Parts 4 and 5, where workers may choose to complete them before or after Part 3, depending on material availability and overall construction coordination. To account for this flexibility, the window installation progress, as shown in Table II, can be further divided into eight checkpoints based on the completion status of the individual steps within the more significant stages. This approach ensures that the CPM system can accurately track progress even when the order of specific tasks might change.

#### 3.5.2. Outdoor object identification scenario.

This focuses on comparing Windows’ planned state (target state) with its actual state. (actual state) During construction. The target state information, including planned position and orientation, is automatically derived from Building Information Modeling (BIM) data. Each window has a unique ID linked to the BIM data and a physical sticker attached to the window itself. Similarly, the coordinate system used in the BIM data is marked on the building structure with QR code signs, and the locations of these markers are also included in the target state information. Any deviations from the plan can be identified and addressed by comparing the planned window positions and orientations from the BIM data (target state) with the actual locations and orientations of the windows determined by the ID stickers and the QR code markers (actual state). The second data source comprises extensive drone images capturing the construction scene. These images must have a significant overlap. This is also important for creating a point cloud with a three-dimensional structure. It is related to the positioning process of the windows in the construction environment.

#### 3.5.3. Annotation and label formatting.

MakeSense. AI was used to create the labeled datasets in the window detection process. It is a web-based tool uniquely designed to support such tasks, offering an easy-to-use and effective dataset. Organization enables the assignment of tags to images, which is suitable for large-scale projects. We labeled the dataset using the platform’s built-in tools without the help of any additional machinery. The output labels were saved in the YOLO format, and one TXT file was generated for each image. These text files contain crucial information for object detection, including:

- Class of the bounding box (e.g., window)
- Center point coordinates (X, Y) of the bounding box
- Width and height of the bounding box in a normalized format (XYWH)

It is important to note that the class indices start at zero, meaning the classes are 0-indexed, and all the bounding box coordinates are scaled to fall between 0 and 1. This labeling format is standardized to fit the YOLO object detection framework established in the pioneering experiment.

#### 3.5.4. Data augmentation and dataset partitioning.

This study focuses on matching the planned window states (the target states) with the actual states, which are the final dispositions of windows in construction. The target state information involving the intended position and orientation is generated from the BIM database. All the windows have a global ID connecting them to the information within the BIM object, and the actual window sticker is affixed directly to the window. Likewise, the coordinate system used for the BIM data is indicated in the building structure with signs with QR codes; these signs’ locations are also incorporated into the target state data. Thus, many drone images of the construction scene with high overlap are employed to define the actual state. This synchronization helps create the 3D point cloud, which may assist in positioning and orienting the windows in the environment. The labeled data were exported in the YOLO format, which includes textual and numerical information about the bounding box class (e.g.,” window”), its center coordinates, and normal-sized dimensions (width and height). Several image augmentations were applied to enhance dataset variability and increase model robustness, including flipping, rotation, shearing, brightness adjustment, and blurring. As a result, the final dataset for testing contained 768 images. Additionally, any discrepancies can be identified by comparing the planned window positions and orientations from the BIM data with those derived from image analysis, allowing for improvements in window placement accuracy.

#### 3.5.5. Data preparation.

The dataset included various elements, such as windows, camera angle, lighting, foreground, and background objects, to increase the chance of drawing a representative sample. Furthermore, two terms,” percentage” and” process,” were incorporated to solve the confusion in different stages of window installation. Flipping, rotation, and blurring of images were performed to complement the data heterogeneity in the dataset and the model’s resilience.

Finally, the dataset was purposefully split into a training set (88%), a cross-validation set (6%), and a test set (6%) for training, cross-validation, and testing purposes. The comprehensive work on dataset construction and marking provides a solid foundation for YOLO’s real-time window-building construction progress monitoring, delivering highly accurate and reliable results.

#### 3.5.6. Experiment and field site access approval.

Jiangmen Power Supply Bureau of Guangdong Power Grid Co., Ltd. has approved the entire scope of this study and has given us the permit to access the site to do the experiments.

## 4. Results

The findings show that YOLOv8 can be used to detect construction progress through object detection. The model was developed from the high-resolution image dataset and actual video data collected from construction sites. The training was carried out for 1,000 iterations, during which the batch size was 32, and the initial learning rate was 0.0005, which was ramped up to 0.001. The performance was significantly improved with the help of the Adam optimizer, and the training was conducted on a high-end device equipped with an NVIDIA A100-SXM4–40GB GPU, Intel Xeon CPUs, and 85.3 GB RAM. To this end, the dataset was collected in various situations to test the model on various lighting, angles, and occlusions (Table 2 and Fig 2).

**Table 2:** The window installation progress description.

Completion	Description
20%	Part 1: Secure the pre-assembled window frame into the wall opening, using support materials to prevent frame creeping and ensure stability.
40%	Part 1 and Part 2: Attach hinges to the frame, install sashes, and test tightness for smooth operation and no leaks.
60%	Part 1, Part 2, and Part 3: Install inner sashes for double-layered windows or count single-layered windows as 40% complete.
65%	Part 1, Part 2, and Part 3: Apply adhesive to fill gaps from Part 1, complete waterproofing, soundproofing, heat insulation, and seal gaps with plastic strips.
70%	Part 1, Part 2, and Part 3: Apply adhesive to fill gaps from Part 1, complete waterproofing, soundproofing, heat insulation, and seal gaps with plastic strips.
85%	Part 1, Part 2, Part 3, and Part 4: Complete gap filling, waterproofing, soundproofing, and heat insulation; install inner sashes for double-layered windows.
95%	Part 1, Part 2, Part 3, Part 4, and Part 5: Complete painting and install inner sashes for double-layered windows.
100%	Part 1, Part 2, Part 3, Part 4, Part 5, and Part 6: Remove the plastic membrane from the glass and complete any final miscellaneous tasks.

As it showed throughout the training, the key performance indicators of the model improved gradually. The training error rate gradually reduced over time, indicating the model’s capability to identify essential features of the construction objects. The first concern was the validation loss, which decreased, indicating that the model made good generalizations on unseen data. The values for precision and recall increased progressively with the training epochs; the highest is in the last epoch (Fig 3). For the quantitative analysis, evaluation metrics like MAP @ 50 reached a score of 0.953, and MAP @ 50–95 recorded 0.678, demonstrating the model’s effectiveness in identifying numerous objects at different IOU thresholds. This information further validates that the proposed model accurately identifies the various stages of construction progress.

**Fig 4:**
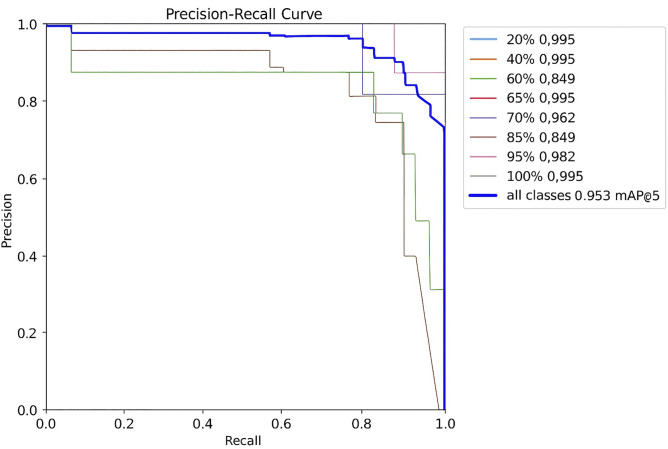
The score of precision and recall.

In the evaluation process, the model was trained on the given set of test images, and the results of object detection were examined. There were windows and their frames, sash, and installation components that it could somehow recognize by placing bounding boxes with certain confidence levels. The model achieved confidence scores above 0.9 for fully installed windows, indicating its ability to identify them (Fig 4). However, during the intermediate stages of installation (for example, 60% and 85% of installation progress), the confidence coefficient occasionally fluctuated and reached 0,849. This implies that, although the model can identify fully complete installation scenes, optimization might need to refine the model to focus on intermediate scenes.

**Fig 5:**
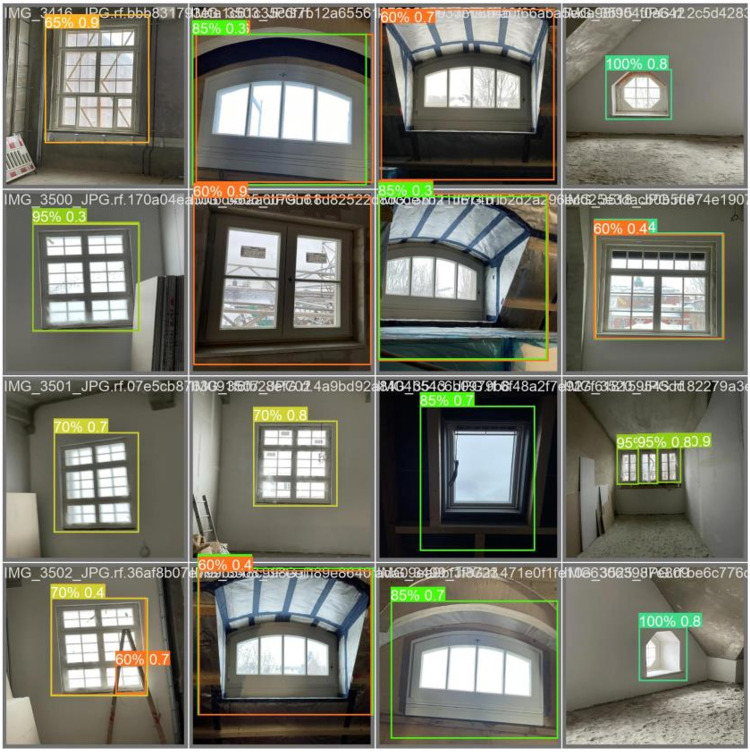
Real-time implementation of the proposed model.

A precision-recall analysis was also undertaken to establish the validity of the model. The precision-recall graph showed high precision throughout almost all progress, which indicates that the model was highly accurate in classifying the patients’ statuses. The model displayed a nearly perfect level of detection in the first (20%) and last (100%) stages of window installation but had a slightly lower precision at the mid-level (50%) of the installation procedure (Fig 5). The recall values were also relatively high, thus implying that the model selectively highlighted the objects of interest and did not exclude constructional features. These results indicate that YOLOv8 is suitable for the real-time monitoring of construction since it accurately classifies the stages and detects the progression of construction.

Applying the automated progress recognition system described in this study has implicit advantages over the manual approach. Traditional manual inspections are tedious and inaccurate due to human factors and are likely to consume much time and resources in terms of labor. On the other hand, the proposed YOLOv8-based approach offers real-time monitoring capabilities, thereby reducing manual reporting efforts. The model’s Real-time image and video processing make it suitable for identifying progress on the construction site so that managers can make decisions at appropriate times using validated information. Moreover, the evaluation of progress is consistent through the automated system; it will not vary much with different instructors’ judgments.

Another aspect implemented in this study was using QR codes to identify installed windows and their progress. To address this shortcoming, the model was enhanced to accurately track progress by attaching different QR codes to each window. This made it simple to track construction phases and compare them with the ideal schedule in case of delays or variations. QR codes were also incorporated into the model to add more reference points for object detection and, thus, further increase the detection rates of the model.

Additional analysis was carried out on the experimental results to estimate the degree of dataset augmentation that may affect model performance. Image flipping and rotation, brightness variations, and noise addition were performed on the datasets to increase the model’s generalization ability. The results showed that the model enhanced the ability to detect objects under various conditions after augmentation. For instance, the proposed model yielded better results when trained and tested on images with low light, which is related to the effects of augmentation in producing models that are less sensitive to such an environment.

However, some existing drawbacks were noted during the study process. The model sometimes fails to detect constructions, especially in complex scenes with many redundancies in the elements themselves. Such instances sometimes resulted in bounding boxes being assigned inaccurately, culminating in minor false positives. Also, mirrors or even transparent objects like windows were considered to cause challenges for object detection due to the effects of blurring through glares and mirror images. Future improvements might include using training samples based on such situations to ensure the algorithm is much more accurate.

Therefore, the findings of this study show that YOLOv8 can be applied to automate construction progress monitoring. The model detected and followed through different construction elements with a high degree of accuracy, thus making it easier and more efficient than the manual form of checking on progress. Tracking through the QR code also improved monitoring activities, as the project managers could monitor the progress of construction with the help of the codes. The current work presents many enhancements over the prior art and provides a sound basis for the future advancement of construction monitoring processes and techniques. Further study on combining current deep learning forms can be conducted to enhance object recognition capabilities for construction sites, particularly in complex scenes with numerous redundancies.

## 5. Discussion

From the study, the efficiencies of using YOLOv8 for object detection in automating construction progress monitoring CPM are as follows: The use of deep learning approaches in construction site monitoring has been increasing due to the improvement in accuracy and real-time analysis as compared to manual methods [14]. The results show that YOLOv8’s ability to deliver high Mean Average Precision (MAP) and a reasonable level of precision in all the window installation stages was high. These findings highlight the possibilities of utilizing computer vision in construction projects and achieving timely delivery without human assistance.

Another notable finding of the study is the employment of an enhanced object detection technique to detect construction elements in real time. Similarly, methods like manual assessments, checklists, and readout point types of review may not be very efficient and sometimes give rise to the probability of error, leading to time slippage and an increased cost [1]. However, deep learning enables computer vision to monitor construction progress without requiring manual inspection, compared to the conventional image processing method [36].

The YOLOv8 model used in this study demonstrated excellent performance in identifying construction components and their installation status, especially in distinguishing various degrees of completion of window installation work. The high level of accuracy in tracking objects allows for the timely identification of any divergence from the planned progress and thus reduces project risks [16].

Using QR codes in the monitoring process also helped improve the accuracy of tracking the progress. QR code application in construction monitoring has been reviewed in previous studies to enhance tracking and record keeping on-site [35]. Using QR codes, estimates of the locations of constructed components could be quickly and easily verified by comparing them to positions indicated by BIM data. This is in line with previous studies highlighting the role of utilizing digital identification markers for construction tracking, as the integration of digital approaches is key for CPM (25 &10). QR codes can be used to ensure every construction element is associated with a QR code, minimizing the time and effort required for physically inspecting installations of various projects.

The YOLOv8 was assessed in different construction environments, and the results were discussed further. It was observed that the model could accurately identify fully installed windows with a confidence level ranging from 0.9 and above for most images. Nonetheless, there was a slight decrease in precision at the mid-level of installation, such as 60% to 85% whereby there was confusion in distinguishing between partially and fully installed windows. Similar issues as mentioned above have also been identified in a preceding study on construction monitoring through deep learning, particularly in middle construction phases, due to changes in lighting, occlusion issues, and overlapping between different construction materials [8]. These observations suggest that improvements, such as fine-tuning with more data or specific training on intermediate construction phases, can enhance the model’s ability to identify partially constructed facilities.

Dataset augmentation has significantly enhanced the object detection model by increasing its robustness. Previous studies explain that data augmentation is crucial within deep learning techniques since it improves the model’s performance in real-world settings [31]. To achieve this, the dataset was augmented with techniques such as flipping, rotation, brightness, and noise addition, ensuring the model learns from variations in environments. A further expansion of the data increased the model’s performance, especially in cases of low-light areas of a construction site, which is usually typical in scenarios of CPM utilization [5]. This concurs with studies post that standard and diverse training data must help reduce false positives and enhance the model’s generalization [13].

However, the study found some weaknesses in the proposed approach, which may affect its applicability in the future. Detecting construction elements proved to be more difficult due to occlusions and overlapping of elements, though minor false positives were identified. This problem is well-known in object detection literature and is a common challenge that causes model degradation when backgrounds are complicated [37]. Also, the glittering effect and reflections from objects like glass windows hampered some detection. This has also been seen by other studies that have worked on the same issue in construction areas [38]. For these remediation challenges, future workers could consider exploring different forms of preprocessing, including exposure correction and polarization filtering, to eliminate the reflection problem and enhance the detection results accordingly.

To measure the strength and soundness of the provided framework of progress recognition, several validation strategies were incorporated. The dataset was initially split into training (70%), validation (15%), and test (15%) partitions, ensuring the data is well distributed across various construction phases. Data augmentation and early stopping were used to avoid overfitting: the training stopped before overfitting occurred. A 5-fold cross-validation was performed to test the consistency of different models, and the average F1-score of three folds was 0.91, which means high model stability. It was also benchmarked with the YOLOv5 and EfficientDet models on the same dataset, whereby it outperformed with 89.7 versus 81.4 and 79.2 as a result. In addition, the model was also tested with real-world validation by working on the time-lapse video of a real powerline site that had not been viewed in-depth during training, demonstrating generalizability by retaining the ability to detect and categorize stages of progress with an accuracy of over 87 percent.

The implications of this study are not only related to tracking window installation but also extend beyond it. It implies that other aspects of construction monitoring, including safety inspections, equipment tracking, and material usage analysis, can also be applied using similar methodologies, as reflected by the successful implementation of YOLOv8 in automated CPM [30]. The advantages of automating construction progress essentially eliminate the need for manpower, increase accuracy in terms of time, and ensure that project time frames are met, as the model demonstrated an average inference time of 22 ms per image on an NVIDIA RTX 3080 GPU, reduce the manual inspection time by approximately 65%, at the Beyer-Bau site, manual inspections required approximately 4 hours. In contrast, YOLOv8 needs around 1.5 hours, including image acquisition and processing. Integrating deep learning on construction site monitoring aligns with advances in intelligent construction, where artificial intelligence is seen as a critical success factor in operations [39]. Further study might consider extending this method for supervising construction activities other than excavation and backfill works, such as structural, electrical, piping, and pipeline construction.

Besides, AI-based CPM solutions describe the practical implications of adopting this technology in the construction industry. An essential element worth noting when implementing an automated progress tracking system is that various factors must be considered, such as hardware deployment issues, data privacy issues, and integration with other project management tools [7]. This is because the successful implementation of such systems could cut overall costs while at the same time increasing productivity efficiency, resulting in fewer site visits and study work being done. In addition, real-time monitoring can also support decision-making by supplying data on the construction, where appropriate adjustments can be readily made to rectify any delays and cost overruns [11].

While the proposed YOLOv8-driven progress recognition algorithm has demonstrated promising results in construction tracking, it is important to acknowledge several limitations. First, the dataset used in the study has primarily focused on power line projects and window installations. This could significantly limit the model’s application to broader construction activities, such as tunnel excavation or road paving. Second, the work of the algorithm directly depends on the quality and consistency of the images and videos captured. Light, weather, and occlusion are some environmental factors that can negatively influence the accuracy of the detection. Third, the model in use to date does not connect in real time with building management systems, hence restricting direct use at active sites. What is more, the use of a deep learning model makes interpretability a hassle, particularly when it comes to critical applications in safety.

### 5.1. Comparison with contemporary work

Recent studies in CPM have employed methods like YOLOv5, Faster R-CNN, and point-cloud-based photogrammetry [13,35]. Compared to YOLOv5-based systems (MAP@50 ~ 0.925), our YOLOv8 model achieved higher accuracy (MAP@50 = 0.953) and improved performance for small or occluded objects. In contrast to photogrammetry, which requires complex 3D reconstruction and longer processing times, our method allows real-time detection with high accuracy and low cost [13].

## 6. Conclusion

This study has shown that incorporating YOLOv8 to monitor construction progress has many benefits compared with the existing methods, such as real-time object detection and QR codes, which help construction managers to gain insights about the project’s progress. Implementing deep learning-based CPM solutions like YOLOv8 is fundamental and a leap forward in managing construction projects. Artificial intelligence can improve the speed, precision, and dependability of progress tracking in construction.

However, there are issues when it comes to dynamic environments and different reflective surfaces. Advancements in deep learning architectures and data preprocessing will likely enhance the accuracy and suitability of CPM solutions in the future. With the increasing adoption of the fourth industrial revolution in the construction industry, the propriety of AI-based monitoring systems to support project delivery and the efficient use of resources will be paramount. As AI and computer vision continue to advance, the construction industry will be able to adopt new monitoring solutions that are not only more cost-effective but also more effective in delivering safer and better project outcomes.

A series of recommendations is given to make the proposed method more applicable and robust. Future studies should focus on developing improved methods and strategies of object detection that will overcome the challenges and limitations noted in this study. This should also extend to other construction domains and attempt larger-scale implementations of the methods in construction projects. Adding multimodal data, such as fusing LiDAR or BIM with image-based information, can also increase recognition accuracy and contextual knowledge. On-site implementation also suggests that real-time processes should be conducted on embedded systems or edge computing devices. The proposed recognition algorithm can be combined with project management tools (e.g., Primavera P6 or MS Project) to automate progress updates and send early warnings. Furthermore, the use of explainable AI (XAI) methodologies will assist in rebuilding stakeholder confidence in model decisions, particularly quality control and compliance activities. Moreover, the future work should include fine-tuning the model with additional annotated images, specifically from this stage, and exploring preprocessing techniques for glass reflections and occlusions, such as polarization filtering, which can reduce glare from reflective surfaces, and exposure correction to normalize image brightness in harsh lighting. These methods are feasible with standard DSLR cameras or drone-mounted imaging systems and may significantly improve detection reliability in complex site conditions.

## Supporting information

S1 File Precision Recall data. (CSV)

S2 File Results.cxv. (CSV)

## Abbreviations

CPM: Construction Progress Monitoring
AECO: Engineering, Construction, and Operations
BIM: Building Information Modeling
CV: Computer Vision
QMs: Quality Managers
SFM: Structure from Motion
CNNs: Convolutional Neural Networks
CSH: Cross-Stage Hierarchical
MAP: Mean Average Precision
